# Traditional Uses, Bioactive Compounds, and New Findings on Pharmacological, Nutritional, Cosmetic and Biotechnology Utility of *Capsella bursa-pastoris*

**DOI:** 10.3390/nu16244390

**Published:** 2024-12-20

**Authors:** Aleksandra Łukaszyk, Inga Kwiecień, Agnieszka Szopa

**Affiliations:** Department of Medicinal Plant and Mushroom Biotechnology, Faculty of Pharmacy, Medical College, Jagiellonian University, Medyczna 9, 30-688 Cracow, Poland; aleksandra.lukaszyk@student.uj.edu.pl (A.Ł.); inga.kwiecien@uj.edu.pl (I.K.)

**Keywords:** shepherd’s purse, Brassicaceae, phytochemistry, pharmacological properties, nutritional value, new pharmacopeial plants

## Abstract

*Capsella bursa-pastoris* (L.) Medik. (shepherd’s purse) is a medicinal plant recently introduced to European Pharmacopoeia. The main active compounds responsible for the activity profile of the raw material are flavonoids, phenolic acids, amino acids, phytosterols, vitamins and bioelements. This species is known for its properties supporting the functioning of the digestive system and antihemorrhagic properties in the ethnomedicine of Far Eastern countries. Modern research confirms these directions of activity. Additionally, the latest studies prove the anti-inflammatory, antioxidant, antibacterial, antifungal, acetylcholinesterase and anticancer properties and supportive action in the treatment of gynecological diseases. Shepherd’s purse herb also has a strong position as an edible plant due to the growing interest in this plant as “healthy food”. The protective, softening, antibacterial and antioxidant properties of sprout and herb extracts are useful in the production of modern cosmetics. Moreover, *C. bursa-pastoris* is valuable thanks to phytoremediation properties and the numerous practical uses in biotechnology for the creation of new resistant varieties of crop plants from the Brassicaceae family.

## 1. Introduction

*Capsella bursa-pastoris* (L.) Medikus (shepherd’s purse) is the perennial plant from the family Brassicaceae well known in many parts of the world as a medicinal and utility plant. This species has been known and used by humans for at least 8000 years. During archaeological research dating to around 5950 B.C., the seeds of *C. bursa-pastoris* were found in the excavations, in the Neolithic settlements at Catalhoyuk in Turkey [1].

*C. bursa-pastoris* was used medicinally as a hemostatic, vaso-constrictor and antipyretic agent and to treat edema, hypertension, kidney and nerve disorders. This herb by ethnopharmacological indications exerted anti-inflammatory, antioxidant, cardiovascular, antimicrobial, hepatoprotective, sedative, anticancer and other pharmacological effects [2,3]. The pharmacological potential of *C. bursa-pastoris* is determined by the specific chemical composition. Current research has shown the presence of metabolites from flavonoids, phenolics acids, biogenic amines, alkaloids, vitamins and elements as well as the essential oil. *C. bursa-pastoris* has successfully been used in traditional and unofficial therapy.

The monograph of *C. bursa-pastoris* can be found in the Pharmacopoea Genevensis of 1780 (under the name *Thlaspi Bursa-Pastoris* Linn or *Bursa Pastoris* Le Tabouret), as well as in the Pharmacopoea Parisiensis of 1758 (*Bursa Pastoris*). In addition, the herb of this species has a monograph *Bursae pastoris Herba*, published by Commission E, and since 2014, it has been included in the Deutscher Arzneimittel Codex [4]. In 2023, *C. bursa-pastoris* (*Bursae pastoris herba*) was introduced into the European Pharmacopoeia 11th edition [5].

Moreover, since 2011, the plant has had an herbal monograph from the Committee on Herbal Medicinal Products (HMPC) of the European Medicines Agency (EMA). According to this document, *C. bursa-pastoris herba* is recommended as the remedy that reduces heavy menstrual bleeding with regular menstrual cycles in women [6]. *C. bursa-pastoris* has an affirmative opinion in CosIng (Cosmetics Ingredients) [7].

In the scientific literature, there are only two review publications on *C. bursa-pastoris* from 2015 [2] and 2021 [8] (without open access). So far, the latest knowledge on this species has not been summarized in a professional manner based on the latest scientific research. This review aims to systematize knowledge about this species and promote *C. bursa-pastoris* as an important herbal medicine in pharmacy, nutrition and cosmetology. The paper covers the literature pertaining to the biology, phytochemistry, ethnopharmacology, therapeutic potential, biotechnology, usage in cosmetology and phytoremediation of *C. bursa-pastoris* indexed on scientific databases (Google Scholar, PubMed, Scopus, Web of Science, Wiley online library) and books.

## 2. Morphology, Ecology and Distribution

**Figure 1:**
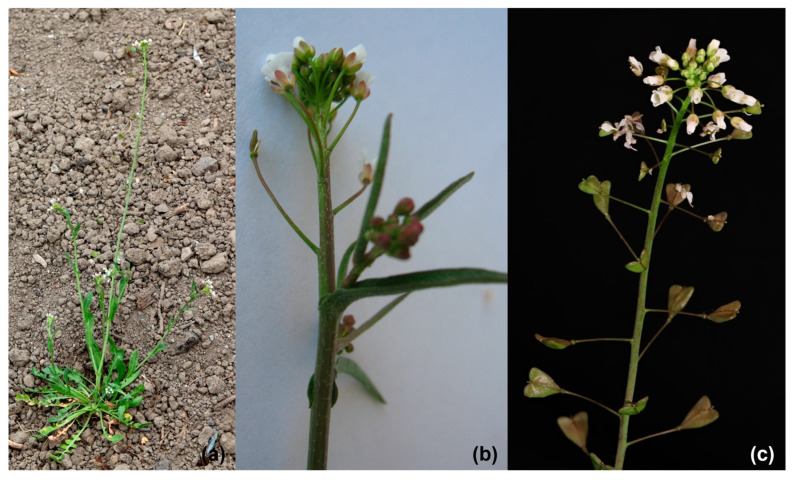
*Capsella bursa-pastoris* plant (**a**); inflorescence shoot with cauline leaves (**b**); inflorescence shoot with flowers and heart-shaped siliques (**c**).

*Capsella bursa-pastoris* is an annual plant, glabrous or slightly hairy, reaching up to 60 cm in height. The species is characterized by a large variability, depending on environmental conditions, as well as the season and the age of the plant. It is a single, white taproot, often forking at a depth of about 10–25 cm, depending on the soil type. Lower leaves are large, sinuate–serrate, serrate, oblong–lanceolate, pinnate or rarely entire, narrowed into a petiole. The stem leaves are smaller, sessile, lanceolate, oblong, sagittate at the base. Corolla is composed of four white or pink petals, which are gathered in a racemose inflorescence. The sepals and petals of the corolla are arranged crosswise as in other species of the Brassicaceae family. A single, upper pistil 0.5 mm long is surrounded by two shorter and four longer free stamens. Flowers are radiant and small. Plants are self-pollinating or insect-pollinating. Fruit is a heart-shaped, triangular silique, in which there are 10–20 oval, small and brown seeds (Figure 1). In colder regions, the plant blooms from March to April and the fruit appears from April to July, while in temperate climates, it blooms from March to October and the fruit ripens after about a month [9,10]. *C. bursa-pastoris* is obtained by sowing seeds in spring in partial shade or in sunny places on airy soils, and once sown, it then spreads itself [11]. The leaves can be harvested one month after sowing [12].

The genus *Capsella* belongs to the family Brassicaceae. The widespread *C. bursa-pastoris* is allopolyploid. Allopolyploidy is a duplication of genomes following hybridization. *C. bursa-pastoris* originated from the hybridization of the *C. grandiflora*/*rubella* and *C. orientalis* lineages some 100–300 thousand years ago. *C. grandiflora* is a curiously genetically diverse obligate outcrosser which occurs in Albania and northern Greece in the mountains. In contrast, *C. orientalis* occurs in Eastern Europe and in the steppes of Central Asia. This plant is a genetically depauperate selfer. Around the Mediterranean Sea occurs *C. rubella*, a selfer derived from *C. grandiflora* [13].

Species of the *Capsella* genus are often very similar to each other. Despite the existence of morphological diagnostic differences, due to their variability, depending on environmental conditions, it is recommended to use molecular methods for their identification.

**Figure 2:**
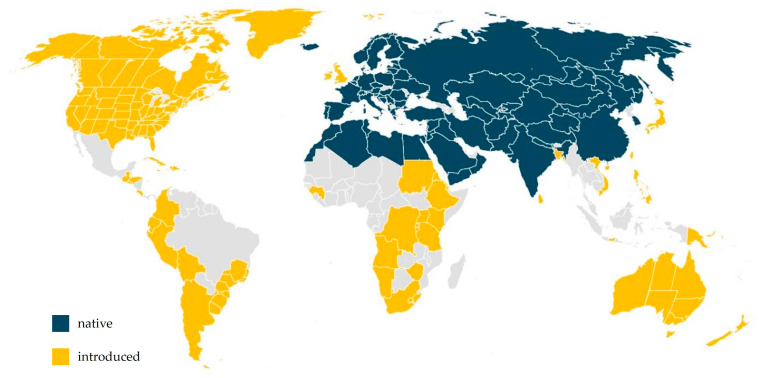
*Capsella bursa-pastoris* distribution map.

*C. bursa-pastoris* is native mainly to Europe, Cyprus, Turkey, Saudi Arabia, Pakistan, Iran, Iraq, India, Azerbaijan, Australia and China. It is also found in North and South America and North Africa (Figure 2) [2,14,15]. It is adapted to temperate, cool and subtropical climates. It can survive at different elevations, from coastal areas to high mountains, and in low and high temperatures [16].

*C. bursa-pastoris* is treated in some regions of Europe as a weed occurring in large numbers in root crop fields, rapeseed, vegetable gardens and cereals. Moreover, it is a common weed of *Coffea* plantations in Central Africa. It is a synanthropic species—both ruderal and segetal. *C. bursa-pastoris* grows along roads, on tracks in yards, on landfills, on fallow land, in gardens, in wall cracks and on rubble. In agricultural crops and gardens, it is a troublesome weed, persisting even in trampled places [17,18].

Shepherd’s purse is a photophilous plant [17], associated with a temperate climate, within which it grows in places with a wide range of temperatures and precipitation variability. It persists even in deserts, in very dry places. Adaptation to dry places includes producing a thick layer of waxes, rolling leaves and increasing their pubescence. *C. bursa-pastoris* is not recorded in permanently humid habitats [10]. Wintering rosettes tolerate frosts down to −12 °C well. In the subtropical and tropical climate zone, it occurs mainly at higher altitudes [9]. *C. bursa-pastoris* grows on various soils, clayey, sandy, stony and shallow, also very compact. It is especially common on heavily fertilized arable soils. With nitrogen deficiencies, very poorly growing starvation forms appear. In turn, the largest plant sizes are achieved on fertile and loose soils [18,19].

Two subspecies, occurring throughout the species’ range-ssp. *bursa patoris* and in southern Bulgaria-ssp. *thracica*—are of recognized taxonomic rank. The endemic plant from Bulgaria deserves to be classified as a species (*Capsella thracica* Velen.) because genetic tests have confirmed that it has a longer pistil neck than *C. bursa-pastoris*. *C. thracica* Velen. is an allopolyploid whose ancestors were *C. grandiflora* (Fauche & Chaub.) Boiss and *C. bursa-pastoris* [20,21].

It is estimated that there are over 250 synonyms for *C. bursa-pastoris*. A large number of taxa have been described due to the extensive range, diversity of habitats occupied and the enormous phenotypic variability of the species [22,23,24,25]. The most common synonyms for the scientific name are *Bursa pastoris*, *Bursa bursa-pastoris* (L.) and *Thlaspi bursa-pastoris* [10,22,23].

This herb has had plenty synonymous, common names in English, like Shepherd’s scrip, Shepherd’s bag, Lady’s purse, Shepherd’s sprout, Rattle pouches, Witches’ pouches, Pick-pocket, Case-weed, Bindweed, Mother’s heart, Pick-purse, Poor man’s parmacet tie, Pepper-and-salt and Sanguinary. In other languages, *C. bursa-pastoris* is known as, e.g., Hirtentäsche (German), Bourse de Pasteur (French), Madakat el Raee, Karmala, Guzman el Raee, Sharbat el Raee, Kess el Raee (Arabic) and tasznik (Polish) [8,25,26,27].

## 3. Chemical Composition

The wide pharmacological spectrum and biological activities of medicinal herbs attract enormous interest in their health benefits. *C. bursa-pastoris* possesses these special activities due to the presence of various phytoconstituents (Table 1). The most important group of secondary metabolites were isolated from the aerial parts of *C. bursa-pastoris*. These include flavonoids and phenolic compounds.

**Figure 3:**
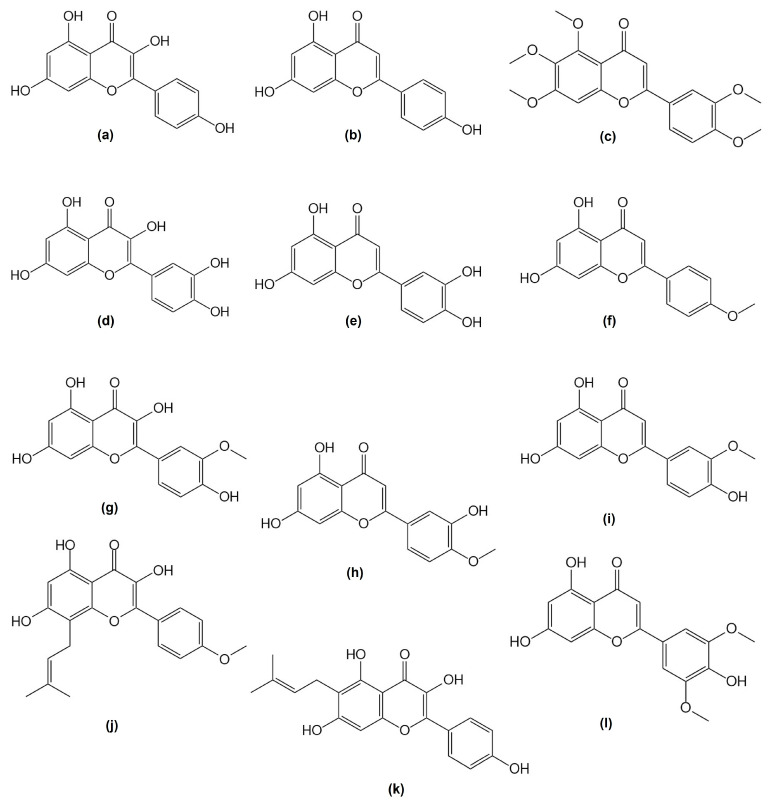
Aglycone structures of flavonoids found in *Capsella bursa-pastoris* extracts: kaempferol (**a**), apigenin (**b**), sinensetin (**c**), quercetin (**d**), luteolin (**e**), acacetin (**f**), isorhamnetin (**g**), diosmetin (**h**), chryseriol (**i**), icaritin (**j**), licoflavonol (**k**) and tricin (**l**).

**Figure 4:**
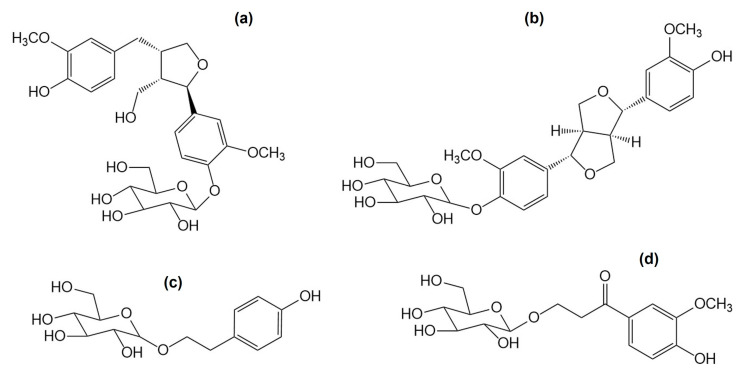
Examples of phenolic glycosides reported in *Capsella bursa-pastoris*: lariciresinol-4′-*O-β*-D-glucoside (**a**); (+)-pinoresinol-*β*-D-glucoside (**b**); salidroside (**c**); *β*-hydroxy-propiovanillone-3-*O-β*-D-glucopyranoside (**d**).

**Figure 5:**
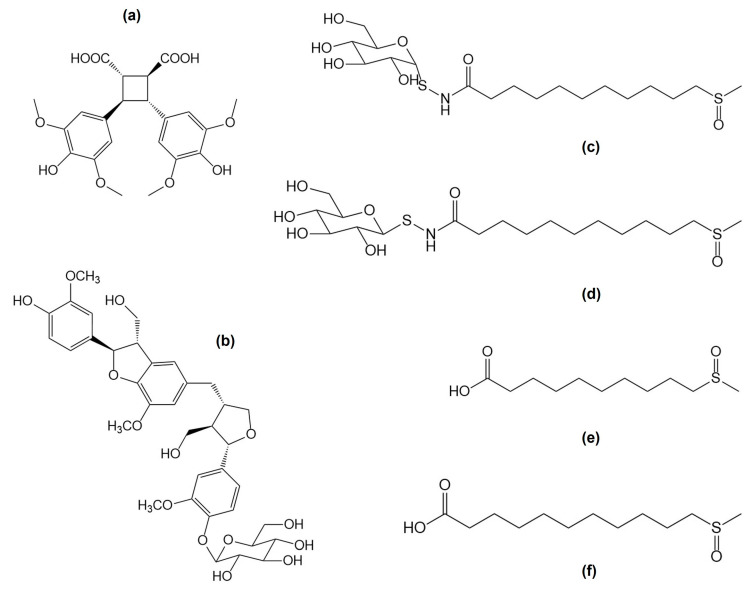
New metabolites recently isolated from the herb—capselic acid A (**a**) and seeds of *Capsella bursa-pastoris*—capselloside (**b**); bursapastoris A (**c**); bursapastoris B (**d**); 10-(methylsulfinyl)decanoic acid (**e**); 11-(methylsulfinyl)undecanoic acid (**f**).

A study on the extracts of *C. bursa-pastoris* confirmed a high content of flavonoids: kaempferol-3-*O*-rutinoside, quercetin-3-*O*-glucoside, quercetin-6-*C*-glucoside, kaempferol and quercetin (Figure 3) [28]. An important chemical group of *C. bursa-pastoris* are phenolic glycosides (+)-pinoresinol-β-D-glucoside, β-hydroxy-propiovanillone 3-*O*-β-D-glucopyranoside and capselloside (Figure 4 and Figure 5) [35].

Analyses of *C. bursa-pastoris* herb extracts have also detected phytosterols. The highest content of β-sitosterol is observed, followed by campesterol and stigmasta-4-en-3-one [28]. Amino acids were isolated from the aerial parts of this species. Tyrosine and arginine were the salient amino acid in extracts [28].

Another group of secondary metabolites are fatty acids. The share of saturated acids is lower than that of unsaturated ones. The palmitic, linolenic and linoleic acids predominate [31]. From organic acid, the highest content indicated is of quinic acid, malic acid and citric acid. Moreover, a characteristic compound of this species, capsellic acid A, was isolated (Figure 5) [28,32].

In addition, *C. bursa-pastoris* has been found to contain essential oil with such compounds: 1-dimethylcyclopentane, ethyl linoleate, palmitic acid and phytane [37].

Organosulfur compounds were identified in extracts from *C. bursa-pastoris* seeds. The presence of bursapastoris A, bursapastoris B, 10-(methylsulfinyl)decanoic acid and 11-(methylsulfinyl)undecanoic acid was confirmed (Figure 5) [34].

*C. bursa-pastoris* is rich with some vitamins like vitamin A, thiamine, riboflavin, niacin, vitamin C), macroelements (Na, Ca, K) and microelements (Fe, Co, Mn, Zn, Cu, Pb) [2,36].

The literature data also indicate the presence of biogenic amines, such as choline (up to 1%), acetylcholine and histamine [38], as well as a peptide with an effect similar to oxytocin [39]. However, the occurrence of these compounds is sometimes questioned [39,40]. Moreover, other active substances found in *C. bursa-pastoris* are hysopine, inositol and the unstable alkaloid bursine [41].

## 4. Ethnopharmacological Indications

The older data on *C. bursa-pastoris* date back to ancient times. Hippocrates (460–370 B.C.) recognized it as a “uterine agent” [42]. However, Pedanius Dioscorides (40–90) and Claudius Galenus (130–200) believed that its fruits have choleretic properties and were used to treat sciatica and internal ulcers. Paracelsus (1493–1541), in turn, treated it as an astringent, decongestant, suppressant of exudates and antihemorrhagic [43].

*C. bursa-pastoris* has been known and used in ethnopharmacy for centuries [8]. The plant has been used in traditional medicine by Japanese and Chinese people for increasing urine output, lowering elevated body temperatures and stopping bleeding. This species has been used in Korea for the treatment of edema and hypertension. In the Altai, *C. bursa-pastoris* has been used for gastritis, dysentery, bleeding, malaria, tuberculosis, heart disease, venereal diseases, metabolic disorders, liver problems and vomiting. In Tibetan medicine, this herb has been used to treat lung, kidney and nerve disorders [8,9].

The herbal raw material of *C. bursa-pastoris* is mainly herb (*Bursae-pastoris herba*) [40,44,45,46] and liquid extract (*Bursae-pastoris extractum fluidum*) [41,46]. Currently, fresh juices squeezed from plants, cold extracts from dried herbs (30–50 mg per 1 L of boiled water) or infusions (20 g per 250 mL of boiling water) are used [41]. They are also available in the form of tincture (*Tinctura bursae from fresh herbs*), dried herb [40] and tablets [39]. A strong decoction is used for uterine bleeding. Two servings consist of a glass of water with a heaped spoon of herb with the addition of barberry, lemon or currant juice and should be used 3–4 times a day. However, the liquid extract should be taken 4–7 times a day, about 40 drops in a glass of water with sour juice [46].

## 5. Applications in Homeopathy

*C. bursa-pastoris* is used in homeopathy. It is mainly an antihemorrhagic remedy. It is used for chronic neuralgia, pain between the shoulder blades and general bruised soreness. The recommended dose of the tincture is up to the sixth strength (Table 2) [47,48].

## 6. Bioactivity and Current Pharmacological Applications

### 6.1. Anti-Inflammatory Activity

The anti-inflammatory activity of eight isolated phenolic glycosides from the aerial part of *Capsella bursa-pastoris* was assessed by measuring the level of nitric oxide (NO) in microglial cells of BV-2 mice. These cells were stimulated with lipopolysaccharide (LPS). The study proved that NO production was significantly inhibited by (+)-pinoresinol-β-D-glucoside (IC_50_ = 17.80 μM). However, the production of the inflammatory mediator was moderately inhibited by β-hydroxy-propiovanillone 3-*O*-β-D-glucopyranoside [35].

The study showed that luteolin isolated from *C. bursa-pastoris* significantly inhibited NO production in LPS-activated BV-2 microglial cells (IC_50_ = 9.70 μM). In turn, 1-*O*-(lauroyl)glycerol and methyl-1-thio-β-D-glucopyranosyl disulfide moderately inhibited the production of nitric oxide (IC_50_ = 32.60 μM and IC_50_ = 44.10 μM, respectively) [49].

Another study was performed on RAW 264.7 cell lines, which were also stimulated with LPS. The study showed that the isolated compounds 10-methylsulfinyl-decanamide and 11-methylsulfinyl-undecanamide have a potential effect on the release of NO. This mechanism involves reducing the mRNA expression levels of cytokines cyclooxygenase-2 (COX-2), inducible NO synthase (iNOS) and interleukin 6 (IL-6) [36]. 

### 6.2. Antioxidant Potential

Quercetin and capsellic acid A isolated from the seeds of *C. bursa-pastoris* are compounds with strong antioxidant activities. In the study, the scavenging rate of 2,2-diphenyl-1-picrylhydrazyl (DPPH) radicals was 94.44% and 91.66%, respectively. It was proven that their activity was similar to that of vitamin C. Isorhamnetin and descurainoside moderately scavenged DPPH radicals (scavenging degree = 76.83% and 69.03%, respectively). However, quercetin-3-*O*-β-D-glucopyranosyl-7-*O*-α-L-rhamnopyranoside and pinoresinol-4-sulfate were characterized by low antioxidant activity. Their DPPH radical scavenging rates were 55.48% and 56.95% [31]. In another study, both methanol and chloroform extracts from the whole plant of *C. bursa-pastoris* showed significant antioxidant potential. The ability to scavenge DPPH free radicals was higher in the chloroform extract, IC_50_ = 235.37 μg/mL [50]. The antioxidant activity of *C. bursa-pastoris* was confirmed by the ABTS assay. The in vitro value was IC_50_ = 66.14 μg/mL in water extract from the dry part of the plant [51].

It was proven that *C. bursa-pastoris* herb methanol extract (MeOH) best inhibited the lipid-peroxyl radical (LOO^•^), EC_50_ = 600.46 μg/mL. However, the methanol–water (1:1) extract (MeOH/H_2_O) was more effective against ^•^NO (EC_25_ = 0.20 μg/mL), O_2_^•−^ (EC_50_ = 167.60 μg/mL) and DPPH^•^ radicals (EC_50_ = 420.96 μg/mL) The study showed that the extracts from *C. bursa-pastoris* have a high ability to scavenge free radicals [28].

The antioxidant capacity determined by FRAP (ferric reducing antioxidant power) assay shows that isorhamnetin, quercetin, descurainoside, pinoresinol-4-sulfate and capsellic acid A isolated from dried seeds of *C. bursa-pastoris* had a high iron-reducing antioxidant capacity (FeSO_4_ equivalence value was 1.19–4.03 mM). The obtained results were similar or even higher compared to the positive control trolox [31].

However, essential oil from the aerial part of *C. bursa-pastoris* was proven to not be a good antioxidant agent. The amount of essential oil needed to inhibit 50% of DPPH radicals (EC_50_) was 100.17 mg/mL and 0.3 mg/mL for the reference antioxidant BHT [32].

### 6.3. Antimicrobial Activity

Scientific studies prove the effectiveness of *C. bursa-pastoris* extracts in inhibiting the growth of Gram-positive and Gram-negative bacteria (Table 3).

To test the antibacterial properties, nine strains of bacteria were used: *Bacillus cereus, Enterococcus faecalis, Escherichia coli, Micrococcus luteus, Proteus mirabilis, Pseudomonas aeruginosa, Salmonella typhimurium, Staphylococcus aureus* and *Staphylococcus epidermidis*. The evaluation of the extracts was made by calculating the minimum inhibitory concentration (MIC). For all Gram-positive bacteria, the MIC value was below 32.00 mg/mL in the MeOH/H_2_O (1:1) extract. The same value was recorded in the MeOH extract for *M. luteus* and *S. epidermidis*. Therefore, the MeOH/H_2_O extract of *C. bursa-pastoris* showed more effective antibacterial activity [28].

The antibacterial activity of water (hot) and ethanol extracts from dried herb of *C. bursa-pastoris* against Gram-positive bacteria (*Enterococcus fecalis*, *Staphylococcus aureus*) and Gram-negative bacteria (*Acinitobacter bumani*, *Escherichia coli*, *Klebsiella pneumoniae*, *Proteus vulgaris*, *Pseudomonas aeruginosa* and *Serratia marcescens*) was tested. The MBC and MIC values of cold water extract were active against most Gram-negative bacteria. Their MIC value ranged from 1200 µg/mL to 2400 µg/mL and MBC ranged from 2400 µg/mL to 4800 µg/mL. Against *K. pneumonia* and *P. aeruginosa*, the MIC was 1200 µg/mL. However, against *S. marcescence* and *E. coli*, the MIC and MBC values were 2400 µg/mL and 4800 µg/mL, respectively. Hot water extract from *C. bursa-pastoris* was active only against *A. bumanii*. The MIC and MBC ranges were 2400 µg/mL and 4800 µg/mL, respectively. On the other hand, for the alcohol extract, the MIC value for *P. aeruginosa* and *K. pneumonia* was 1200 µg/mL, and the MBC value was 2400 µg/mL [52].

The isolated sulforaphane-containing solution (SCS) from *C. bursa-pastoris* inhibited the growth of *Bacillus anthracis* and vancomycin-resistant *Enterococci*. The MIC value was 250 µg/mL and 1000 µg/mL, respectively [53].

**Table 3:** Comparison of minimal inhibitory concentration (MIC) [mg/mL] of *Capsella bursa-pastoris* extracts tested against Gram-positive and Gram-negative bacteria.

Strains	MIC Value and Extract Tested	References
**Gram-positive bacteria**		
*Bacillus anthracis*	0.25 (sulforaphane-containing solution from *C. bursa-pastoris*)	[53]
*Bacillus cereus*	32 (MeOH/H_2_O extract)	[28]
*Enterococcus faecalis*	32 (MeOH/H_2_O extract)	[28]
*Enterococcus* sp. (vancomycin-resistant)	1 (sulforaphane-containing solution from *C. bursa-pastoris*)	[53]
*Micrococcus luteus*	32 (MeOH and MeOH/H_2_O extracts)	[28]
*Staphylococcus aureus*	32 (MeOH/H_2_O extract)	[28]
*Staphylococcus epidermidis*	32 (MeOH and MeOH/H_2_O extracts)	[28]
**Gram-negative bacteria**		
*Acinitobacter bumani*	2.4 (hot water extract)	[52]
*Escherichia coli*	>125 (MeOH and MeOH/H_2_O extracts)	[28]
	2.4 (cold water extract)	[52]
*Klebsiella pneumoniae*	1.2 (cold water extract and ethanol extracts)	[52]
*Pseudomonas aeruginosa*	>125 (MeOH and MeOH/H_2_O extracts)	[28]
	1.2 (cold water extract and ethanol extracts)	[52]
*Proteus mirabilis*	>125 (MeOH and MeOH/H_2_O extracts)	[28]
*Salmonella typhimurium*	>125 (MeOH and MeOH/H_2_O extracts)	[28]

The antifungal and antibiofilm activity of *C. bursa-pastoris* ethanol and methanol extracts against selected *Candida* species (*C. albicans*, *C. dupliniensis C. glabrata*, *C. parapsilosis* and *C. tropicalis*) was demonstrated. The formation zone was 15–18 mm. The root extract showed > 250 mg/L in microdilution tests. Crystal violet (CV) assay showed that reducing the concentration of *C. bursa-pastoris* root extracts significantly increased biofilm formation. The minimum biofilm inhibitor concentration (MBIC) value was 64 mg/L against *C. tropicalis*. On the other hand, ethanol extract from flowers of the tested species had activity against *C. albicans* (15 mm). However, the extract from *C. bursa-pastoris* leaves did not show satisfactory results [54].

### 6.4. Anticholinesterase Activity

The study showed that the dried hexane extract of *C. bursa-pastoris* has the highest inhibitory activity against the acetylcholinesterase (AChE) enzyme (IC_50_ = 7.24 µg/mL) among other extracts of other tested plants. The anti-AChE activity of the extracts was compared with the drug used in Alzheimer’s disease—galantamine (IC_50_ = 0.5 µg/mL) [51]. However, in another study, the MeOH extract and MeOH/H_2_O (1:1) extract of this plant showed high inhibitory activity against AChE (EC_50_ = 909.44 µg/mL and EC_50_ = 3579.41 µg/mL, respectively) [28]. These studies give hope for further additional tests that could confirm the possible effectiveness of *C. bursa-pastorsis* in the prevention and treatment of neurodegenerative diseases.

### 6.5. Hepatoprotective and Antihypercholesterolemic Activity

A moderate hepatoprotective effect of the isolated compounds 4′,7-dihydroxy-5-hydroxymethyl-6,8-diprenylflavonoid, chrysoeriol-7-*O*-β-D-glucopyranoside, sinensetin and 6,8-diprenylgalangin from the aerial parts of *C. bursa-pastoris* was confirmed. The study was performed using the MTT colorimetric method on WB-F344 rat hepatic epithelial stem-like cells. Cell damage was induced by D-galactosamine [3].

The mechanism of the hepatoprotective effect of the ethanol extract from *C. bursa-pastoris* containing 17.5 mg icaritin per kilogram of the extract and icaritin on HepG2 cells and in obese mice was investigated. The extract was shown to significantly reduce serum LDL and total cholesterol levels in obese mice by reducing PCSK9 gene expression. *C. bursa-pastoris* extract and the contained icaritin suppressed HNF-1α and SREBP2 transcription factors, which resulted in a reduction in the intracellular level of the low-density lipoprotein receptor (LDLR) and PCSK9. The extracellular level of PCSK9 was suppressed significantly by icaritin in HepG2 cells. The study showed that icaritin and *C. bursa-pastoris* extract can be used alternatively in hypercholesterolemic therapy [55].

### 6.6. Anticancer Activity

The anticancer activity of extract (dried herb with distilled water) from *C. bursa-pastoris* against cervical cancer cells (human WISH cell line) was confirmed. The study used a method involving inhibition and percentage determination of cancerous cell lines. In total, 67% of tumor cells were inhibited at the minimum concentration of 0.016 g/mL, while at 0.031–0.125 g/mL, the line survival was 7% [56].

The study demonstrated the potential of hot water extract from *C. bursa-pastoris* on alleviating doxorubicin (DOX)-induced cardiotoxicity (DICT). The experiment was performed on MDA-MB-231 human breast cancer cells and H9c2 rat cardiomyocytes. The tested extract did not affect DOX-induced MDA-MB-231 cell death but inhibited DOX-induced H9c2 cell death. The mechanism was a dose-dependent increase in superoxide dismutase (SOD) levels, which decreased the production of reactive oxygen species (ROS) and increased oxygen consumption in rat myoblasts. Administration of *C. bursa-pastoris* extract with DOX to C57BL/6 mice improved RR interval, heart rate and QT and ST prolongation. Moreover, serum lactate dehydrogenase (LDH) and creatinine kinase (CK) levels decreased. Researchers suspect isoorientin and luteolin-7-*O*-glucoside, which were the main compounds among the seven identified flavonoids, to have a principial role in the protective effect [57].

### 6.7. Effect on the Circulatory System

The antihypertensive effect of hot water extract from *C. bursa-pastoris* was shown by angiotensin converting enzyme (ACE) inhibitory activity, which was at a level of 33.95% [58]. Additional studies confirming the possible effectiveness of *C. bursa-pastoris* in lowering blood pressure should be conducted.

### 6.8. Diseases of the Gastrointestinal Tract

A study was conducted on patients, the aim of which was to examine the effect of a tea preparation with *C. bursa-pastoris* (200 mL a day) on the occurrence of symptoms of hemorrhoidal disease. After 3 months of use, fewer difficulties and bleeding during defecation were observed in the study group than in the control group. Lower visual analogue scale (VAS) pain scores were also noted in this group of patients. The obtained results suggest that the use of *C. bursa-pastoris* herbal tea may reduce the symptoms caused by hemorrhoids [59].

### 6.9. Gynecological Diseases

The clinical study assessed the effect of alcoholic extract of *C. bursa-pastoris* on the control of heavy menstrual bleeding and quality of life in people with uterine leiomyoma. The study group received a capsule filled with 350 mg of dried extract twice a day for 3 months, and the control group received a capsule filled only with 150 mg of starch, also twice a day. The mean Pictorial Blood Assessment Chart (PBAC) was initially 464.00 and after three months 323.82 in the study group. However, in the control group, the mean decreased from 445.92 to 214.36. The study showed that in both groups, there was an improvement in the quality of life during menstruation and a shortened average duration of menstrual bleeding. There was no significant difference between the groups. Therefore, the researchers suggested the need to conduct a study in one specific type of uterine leiomyoma and with a larger group of patients [60].

Another clinical study conducted to determine the effect of hydroalcoholic extracts of *C. bursa-pastoris* on heavy menstrual bleeding showed better results [61]. The intervention group took two capsules (500 mg) of mefenamic acid every 8 h and two capsules containing 640 mg *C. bursa-pastoris* extract (equal to 5 g of the herb) every 12 h. Patients in the control group took mefenamic acid and placebo (starch). The intervention included three menstrual cycles. The assessment was made by measuring PBAC and the number of bleeding days. Capsules with *C. bursa-pastoris* extract were proven to be effective in reducing the amount of menstrual bleeding [61].

## 7. Nutritional Importance

Research suggests that *C. bursa-pastoris* may be used as a functional food. It can be used as a leafy vegetable for consumption due to its good source of microelements, protein and energy [50,53]. Nevertheless, as herbal dietary supplement *C. bursa-pastoris* is not regulated by the Food and Drug Administration (FDA) and the European Food Safety Authority (EFSA).

Raw or cooked leaves of the mature *C. bursa-pastoris* plant have a peppery [12], bitter [47] and burning [62] taste [63]. However, young leaves, harvested before the plant flowers, have a milder taste [52]. Both can be added to cottage cheese, sauces, salads [47] and sandwiches and also made into juices and smoothies [63]. The fruit of this plant also has a peppery taste and can be used to season dishes [11], while the root is used as an equivalent to ginger [12,62].

*C. bursa-pastoris* is eaten pickled or as a salad with mashed potatoes. In Pshavi (Georgia), the leaves and young shoots are eaten. In Khevi (Georgia), *C. bursa-pastoris* is mixed with other plants to prepare the traditional Georgian dish pkhali [8]. In Hayrat (Turkey) and Kalkandere, the stem of this plant is eaten as a meal for the intestines [64]. Locally, *C. bursa-pastoris* is called oban antas in Ordu and Samsun Cities. Its above-ground part is used to produce flour products and is also eaten raw [65]. In the Caucasus, young leaves of this plant are added to soups.

In Japan, *C. bursa-pastoris* is one of the seven ingredients of a mixture of “spring herbs” (haru no nanakusa). In China, it is used as a filling for dumplings [62].

Moreover, *C. bursa-pastoris* is a melliferous and oil-producing plant [41]. Honey from its flowers has a pleasant taste and is light yellow in color [41]. The *C. bursa-pastoris* seeds are used to produce edible oil [12,41]. The energy value of *C. bursa-pastoris* leaves is 67 kcal [2].

## 8. Cosmetological Applications

In dermatology, *C. bursa-pastoris* is used to treat eczema [66]. Moreover, the German Institute for Pharmaceuticals and Medicines has registered preparations containing *C. bursa-pastoris* and recommends it for the treatment of wound and skin diseases [67].

The CosIng Database includes extracts from *C. bursa-pastoris* along with a description of their functions [7]. One of the three products is *Bacillus/Monascus/Capsella Bursa Pastoris* Leaf/rice bran ferment filtrate, which is produced by the fermentation of *C. bursa-pastoris* leaves and *Oryza Sativa* (Rice) bran by the microorganisms *Monascus* and *Bacillus.* Products with *C. bursa-pastoris* are also included in the Cosmetics Europe Database (COSMILE Europe) (Table 4) [6,68]. Moreover, the catalogue of cosmetic products available in the pharmacy (Dermindex) includes preparations containing *C. bursa-pastoris* [69].

There are cosmetics available on the market containing the extract of this species in the form of creams, body and face lotions. They also occur as bath salts, body oils for children and cooling or warming preparations [70].

## 9. Phytoremediation Ability

The development of industry has led to the contamination of water and soil by sewage containing metal and non-metal compounds. That is why many scientists focus their research on finding plants that could effectively participate in cleaning the environment from post-industrial waste or heavy metals. Such studies have also been conducted on *C. bursa-pastoris*.

*C. bursa-pastoris* showed high bioassimilation accumulation of the metals like copper, cadmium, zinc and iron, which was proven based on tests conducted on plants collected in western Iraq [71]. A study was conducted to determine the ability of minced *C. bursa-pastoris* root with peroxide to phytoremediate soil contaminated with toxic compounds derived from herbicides 2,4-dichlorophenol (2,4-DCP). 2,4-DCP was maximally removed from the soil within 10 min after treatment [72]. Another study proved the ability to bioaccumulate cadmium (Cd). When the Cd concentration in the soil was 50 mg/kg, the content of this metal in *C. bursa-pastoris* shoots was above 100 mg/kg [73]. Moreover, in the studies conducted on plants from Brassicaceae, it was proven that *C. bursa-pastoris* showed the best bioaccumulation for Cr (114.59 ppm) and Cu (95.58 ppm) compared to the other tested species [74].

## 10. Application in Biotechnology

The leading research direction in *C. bursa-pastoris* biotechnology studies is genetic transformation. It is now considered a model species for the study of embryogenesis [75]. Using the cDNA-AFLP technique, 231 genes expressed during the embryonic development of *C. bursa-pastoris* were identified. Many of them were related to metabolism, transcription and oxidative stress, as well as protection against binding proteins and diseases [76].

*C. bursa-pastoris* genome nowadays is used to modify varieties of Brassicaceae crop plants. It is used for hybridization with species of the *Brassica* genus in order to transfer genes determining the low content of erucic acid and glucosinolates, as well as resistance to *Sclerotinia sclerotiorum* [77]. Low temperature induces *C. bursa-pastoris* genes that are responsible for its tolerance of saline habitats and frost resistance [78,79]. As a result, transgenic tobacco varieties with greater frost resistance are obtained [79].

A study was carried out in which inactive protoplasts of *Brassica oleracea* were hybridized with protoplasts of *C. bursa-pastoris* in order to increase the activity of this commercial species. Intertribal somatic hybrids were generated from iodoacetate-treated *B. oleracea* protoplasts via polyethylene glycol-mediated fusion of untreated *C. bursa-pastoris* protoplasts. Thanks to this, *B. oleracea* had genes characteristic of *C. bursa-pastoris*, such as frost tolerance and probably drought and salt tolerance [78,80,81]. Moreover, resistance to *Alternaria brassicicola* has been proven as a result of hybridization with protoplasts of *B. oleracea*. The obtained tetraploid hybrids rooted easily after being transferred to the soil however, unlike diploid hybrids, their flowers were sterile. [82].

Another study was performed using suspension culture of *C. bursa-pastoris*. Embryogenesis was observed in microcolonies composed of protoplasts but germinated without growth regulators. As a result, easily rooted shoots were obtained, which developed into seedlings very quickly. The study proved that *C. bursa-pastoris* is a good gene source for the introgression of cultivated *Brassica* plants through somatic hybridization and protoplast manipulation [83].

Using leaves, cotyledons, petioles and hypocotyls of *C. bursa-pastoris* as explants, an experiment was performed to investigate how different media influence the induction and subculture of callus, the appearance of callus-induced explants and plant regeneration. It was found that hypocotyl showed the best characteristics for subculture and callus induction. In turn, the preferred medium for plant regeneration for this explant was Murashige and Skoog (MS) medium with the addition of growth regulators: 2–3 mg/L 6-benzylaminopurine (6-BA) and 0.2–0.6 mg/L 1-naphthylacetic acid (NAA). The effect on callus re-differentiation was also confirmed by using a concentration of 2,4-D in its culture [84].

*C. bursa-pastoris* embryos growing individually on MS medium or in in vitro culture ovules are characterized by slow differentiation and low growth compared to in situ embryos. Compared to in vitro embryos, egg tissue induces growth and survival at a similar level but has a different impact on differentiation [85]. Older and early embryos of *C. bursa-pastoris* were cultured on semi-liquid medium and with access to light for only 12 h. Cultures growing continuously without light initiated the primary root system and lateral roots compared to those growing in the light. On the other hand, this process was not inhibited in embryos over 1000 µm long. However, a relationship was found between the growth rate of *C. bursa-pastoris* embryos and their initial length. Growing in the light was characterized by better growth. After adding adenine sulfate, kinetin and indole acetic acid to the medium, the development of spherical embryos (less than 80 µL in length) was observed in vitro [86]. The 500 µm long *C. bursa-pastoris* ovules cultured in vitro developed differently than in situ. The embryo showed growth, while the endosperm was present in both cellular and liquid form in situ, but then it showed slower growth. On the other hand, to ensure its development was even, an egg tissue was grown that was attached to the placenta. This method improved their growth and survival, which is why it is used to develop very small embryos [87]. The mineral solution used affects the survival of immature *C. bursa-pastoris* embryos cultured in vitro. By adding mineral substances, it causes its growth, most likely as a result of substances necessary for embryonic development entering the medium through a crack in the suspension. On the other hand, their sudden entry into the suspension reduces the survival of the embryos. The same tendency persists in the case of a single inoculation into the medium [88].

## 11. Activity Against Harmful Insects

Research on *C. bursa-pastoris* conducted in various directions gives hope for its use in many fields. The seeds of *C. bursa-pastoris* secrete mucus on contact with water. This causes mosquito larvae to stick to them and die. The study showed that 27 larvae were attached to one seed. This method is recommended for combating them in small water reservoirs [89]. Moreover, this species was used to exterminate cattle lice and nits in Belarusian lands [90]. This freshly harvested plant can also be used to control bedbugs [91]. Interestingly, the extract of *C. bursa-pastoris* inhibits the corrosion of steel in 1M hydrochloric acid, due to the formation of a film on the metal surface [92].

## 12. Summary

Nowadays, modern dietetics, cosmetology, as well as herbal medicine, apart from searching for new species in the plant world, are rediscovering this well-known species from ethnobotany.

*C. bursa-pastoris* is a species that is quite widespread but not known in all regions of the world. It is undoubtedly a species recognized from the traditional medicine of East Asian countries, where its properties of stopping bleeding and supporting the functioning of the digestive tract, especially the work of the liver, have long been used.

Phytochemical studies indicate that the main compounds responsible for the properties in the raw material are flavonoids, mainly derivatives of kaempferol, quercetin, apigenin and luteolin and phenolic acids derivative of quinic, ferulic and *p*-coumaric acids. In addition, the raw materials contain amino acids, phytosterols, fatty acids, organic acids, vitamins and bioelements, as well as specific compounds from the group of sulfur glycosides.

Modern studies confirm the ethnopharmacological indications for the use of *C. bursa-pastoris* herb. In addition, they prove the effective mechanisms of anti-inflammatory and antioxidant action of the raw material. Effective action was also found in relation to bacteria *A. bumani*, *B. anthracis*, *B. cereus*, *E. faecalis*, *E. coli*, *K. pneumoniae*, *M. luteus*, *P. aeruginosa*, *S. marcescens*, *S. aureus*, *S. epidermidis* and fungi: *C. albicans*, *C. dupliniensis*, *C. glabrata*, *C. parapsilosis* and *C. tropicalis. C. bursa-pastoris* extracts also have confirmed cholesterol-lowering and hepatoprotective actions. Single studies show anticancer action (in relation to cervical cancer cell lines) and cholinesterase-inhibiting properties, as well as a cardioprotective role in doxorubicin treatment. A number of studies also prove the effectiveness of *C. bursa-pastoris* using extracts in the treatment of uterine fibroids and heavy menstrual bleeding.

To sum up, scientific research has consistently confirmed the traditionally known and proven new possible uses of *C. bursa-pastoris*. It confirms the validity of introducing this species as a new medicinal plant in the latest editions of the pharmacopoeia.

## Figures and Tables

**Table 1:** Chemical composition of *Capsella bursa-pastoris*.

Group of Compounds	Compounds	References
Flavonoids	acacetin-7-*O*-β-D-glucopyranoside, apigenin-6-*C*-hexoside-8-*C*-pentoside, chrysoeriol-7-*O*-β-D-glucopyranoside, licoflavonol, sinensetin, icaritin, 6,8-diprenylgalangin, chrysoeriol, chryseoriol-7-*O*-glucoside, diosmetin-7-*O*-triglycoside, isorhamnetin-3-rutinoside, kaempferol, kaempferol-*O*-rhamnoside, kaempferol-*O*-glucoside, kaempferol-3-*O*-β-D-rutinoside, kaempferol-7-*O*-α-L-rhamnopyranoside, kaempferol-3-*O*-β-D-glucopyranosyl-7-*O*-α-L-rhamnopyranoside, kaempferol-3-*O*-rutinoside, luteolin, luteolin 6-*C*-β-glucopyranoside, luteolin-6-*O*-glucoside, luteolin-6-*C*-pentoside-8-*C*-hexoside, 4′,7-dihydroxy-5-hydroxymethyl-8-prenylflavonoid, 4′,7-dihydroxy-5-hydroxymethyl-6,8-diprenylflavonoid, quercetin, quercetin-3-(6-*O*-acetyl-β-glucoside), quercetin-6-*C*-β-D-glucopyranoside, quercetin-3-*O*-β-D–glucopyranoside, quercetin-3-*O*-rutinoside, quercetin-6-*C*-glucoside, quercetin-3-*O*-β-D-glucopyranosyl-7-*O*-α-L-rhamnopyranoside, tricin	[3,8,28,29,30]
Phenolic acids	5-*O*-caffeolyquinic acid, *p*-coumaric acid, 3-*p*-coumaroylquinic acid, 5-*p*-coumaroylquinic acid, 4-*p*-coumaroylquinic acid, 1-*O*-feruloylquinic acid, 5-*O*-feruloylquinic acid	[29]
Amino acids	arginine, asparagine, cysteine, glycine, histidine, isoleucine, leucine, lysine, ornithine, phenylalanine, proline, serine, threonine, tryptophan, tyrosine, valine	[28]
Phytosterols	campesterol, cholesterol, cholest-5-en-3-one, ergosta-4,6,8(14),22-tetraen-3-one, lupeol, β-sitosterol, stigmasterol, stigmasta-4-en-3-one, stigmasta-3,5-dien-7-one	[28]
Fatty acids	arachidic acid, heptadecanoic acid, lauric acid, linoleic acid, myristic acid, oleic acid, palmitic acid, palmitoleic acid, pentadecanoic acid, stearic acid, (*Z*)-6-octadecenoic acid, 9,10-(*Z*)-methylene-hexadecanoic acid, (*Z*)-7-hexadecenoic acid	[28,31]
Organic acids	capsellic acid A, citric acid, fumaric acid, malic acid, oxalic acid, shikimic acid, quinic acid	[28,32]
Sulfur glycosides	10-(methylsulfinyl) decanamide, 11-(methylsulfinyl) undecanamide, bursapastoris A, bursapastoris B	[33,34]
Phenolic glycosides	coniferin, β-hydroxy-propiovanillone-3-*O*-β-D-glucopyranoside, 3-(4-β-D-glucopyranosyloxy-3,5-dimethoxy)-phenyl-2*E*-propanol, salidroside, (+)-pinoresinol-β-D-glucoside, lariciresinol-4′-*O*-β-D-glucoside, *7S*, *8R*, *8′R*-(−)-lariciresinol-4,4′-bis-*O*-glucopyranoside, capselloside, 1-feruloyl-β-D-glucopyranoside	[35]
Vitamins	niacin, riboflavin, thiamine, vitamin A, vitamin C	[2]
Macroelements	Ca, K, Na	[2,36]
Microelements	Co, Cu, Fe, Mn, Pb, Zn	[2,36]

**Table 2:** Indications of *Capsella bursa-pastoris* in homeopathy.

Distinction Based on Gender or Location of the Medical Problem	Disease or Disfunction
Female disorders	metrorrhagia, leucorrhea after and before menses, hemorrhage with violent uterine colic and pain in womb on rising
Male disorders	spermatic cord sensitive to concussion of riding or walking
Head and nose	eyes and face puffy, mouth and lips cracked, tongue white, coated, frontal pain, vertigo, and scaly eruption behind ears, bleeding in nasal operations
Urinary system	chronic cystitis, renal colic, urethritis, Brick-dust sediment, urine heavy, phosphatic, hematuria, dysuria and spasmodic retention accumulation of gravel, renal and vesical irritation

**Table 4:** *Capsella bursa-pastoris* forms used in cosmetic products allowed by COSMILE Europe Database and CosIng Database.

Name	Form	Function
*C. bursa-pastoris* sprout water	aqueous extract from sprouts	protecting the skin against external factors, antioxidant
*C. bursa-pastoris* extract	extract of the whole herb	protecting the skin against external factors
*Bacillus/Monascus/C. bursa-pastoris* leaf/rice bran ferment filtrate	from *C. bursa-pastoris* only leaves	smoothing and softening the skin, antibacterial, protecting the skin against external factors and antioxidant
